# Health impact of the 2008 cold spell on mortality in subtropical China: the climate and health impact national assessment study (CHINAs)

**DOI:** 10.1186/1476-069X-13-60

**Published:** 2014-07-24

**Authors:** Mai Geng Zhou, Li Jun Wang, Tao Liu, Yong Hui Zhang, Hua Liang Lin, Yuan Luo, Jian Peng Xiao, Wei Lin Zeng, Ye Wu Zhang, Xiao Feng Wang, Xin Gu, Shannon Rutherford, Cordia Chu, Wen Jun Ma

**Affiliations:** 1 The National Center for Chronic and Noncommunicable Disease Control and Prevention, Beijing 100050, China; 2 Guangdong Provincial Institute of Public Health, Guangdong Provincial Center for Disease Control and Prevention, Guangzhou 511430, China; 3 Environment and Health, Guangdong Provincial Key Medical Discipline of Twelfth Five-Year Plan, Guangzhou 511430, China; 4 Guangdong Provincial Center for Disease Control and Prevention, Guangzhou 511430, China; 5 Chinese Center for Disease Control and Prevention, Beijing 102206, China; 6 Griffith University, Brisbane 4111, Australia

**Keywords:** Cold spell, Mortality, China, Subtropical, Extreme temperatures

## Abstract

**Background:**

Many studies have investigated heat wave related mortality, but less attention has been given to the health effects of cold spells in the context of global warming. The 2008 cold spell in China provided a unique opportunity to estimate the effects of the 2008 cold spell on mortality in subtropical regions, spatial heterogeneity of the effects, stratification effect and added effects caused by sustained cold days.

**Methods:**

Thirty-six study communities were selected from 15 provinces in subtropical China. Daily mortality and meteorological data were collected for each community from 2006 to 2010. A distributed lag linear non-linear model (DLNM) with a lag structure of up to 27 days was used to analyze the association between the 2008 cold spell and mortality. Multivariate meta-analyses were used to combine the cold effects across each community.

**Results:**

The 2008 cold spell increased mortality by 43.8% (95% CI: 34.8% ~ 53.4%) compared to non-cold spell days with the highest effects in southern and central China. The effects were more pronounced for respiratory mortality (RESP) than for cardiovascular (CVD) or cerebrovascular mortality (CBD), for females more than for males, and for the elderly aged ≥75 years old more than for younger people. Overall, 148,279 excess deaths were attributable to the 2008 cold spell. The cold effect was mainly from extreme low temperatures rather than sustained cold days during this 2008 cold spell.

**Conclusions:**

The 2008 cold spell increased mortality in subtropical China, which was mainly attributable to the low temperature rather than the sustained duration of the cold spell. The cold effects were spatially heterogeneous and modified by individual-specific characteristics such as gender and age.

## Introduction

The Intergovernmental Panel on Climate Change (IPCC) has projected that in the coming decades, extreme weather events will become more frequent and more intense in some parts of the world and such events will impact on health [1]. Generally, the health effects of extreme heat events are acute and some harvesting is observed, but the effects of extreme cold temperatures are generally more prolonged than heat without mortality displacement [2-4]. Due to the projections associated with climate change many more studies have been conducted on health effects of heat waves compared to cold-related health impacts [2,5-11]. Moreover, most previous studies on health effects of cold spells were conducted in temperate climate developed countries with very few in tropical or subtropical regions [2,8,9]. However, the health effects of extreme cold spells may be larger in these warm regions because populations are not acclimatized to cold spells and are unprepared for such events [3].

Traditional quantitative approaches to investigate the health effects of temperature are of 2 types-episode analysis and continuous-temperature time-series analysis [10,11]. Recently, Gasparrini and his colleagues combined the two approaches to investigate heat wave-related mortality, which divided heat effects into two parts: independent contributions of daily temperature occurrences and the effects of exposure to hot temperatures protracted for several days [12]. This approach is helpful to better understand the mechanism of temperature on mortality, and provides more significant information on public health intervention and cold-related burden estimation under projected climate change scenarios. However, few studies used this new approach to investigate the cold spell effects [11].

In January and February 2008, the majority of China, especially fifteen provinces of subtropical southern China experienced a severe continuous cold spell of a long duration, with lower than normal temperatures, heavy precipitation and thick snow deposition. Average air temperature during the cold spell days was 2–4°C lower than that during the same period of neighboring years. This event is considered a once in 50–100 years event. The estimated direct economic losses were more than US $22.3 billion [13,14]. This extreme weather event provides a unique opportunity to assess the health impact of extreme cold spells on populations in subtropical regions (between the Tropic of Cancer and Tropic of Capricorn) [15]. Three previous Chinese studies, two from Shanghai and one from Guangdong Province have reported that the 2008 cold spell significantly increased mortality or morbidity risk [3,16,17]. However, some health issues associated with this event remain unclear. Firstly, these published studies only focused on a single city or province, which prevents an estimate of the total effect of this extreme cold spell on mortality in subtropical China [10]. Secondly, the spatial distribution of effects of this event on mortality across southern China are unknown, and such information is helpful to identify vulnerable regions and populations [18]. Thirdly, whether the duration of extreme low temperatures sustained for several consecutive days imposed an added effect to the independent effects of daily temperature levels is unclear, and as this has been observed in relation to heat wave related effects [12]. This type of analysis is helpful to better understand how it impacts on health. Thus, it is necessary to conduct multi-community studies to comprehensively assess health impacts of the 2008 cold spell in China, which can expand our understanding of the health impacts of cold spell events in subtropical regions. This study aimed to estimate the effects of the 2008 cold spell on mortality in subtropical regions of China, spatial heterogeneity of the effects, stratification effect and added effects caused by sustained cold days.

## Methods

### Study settings

**Figure 1:**
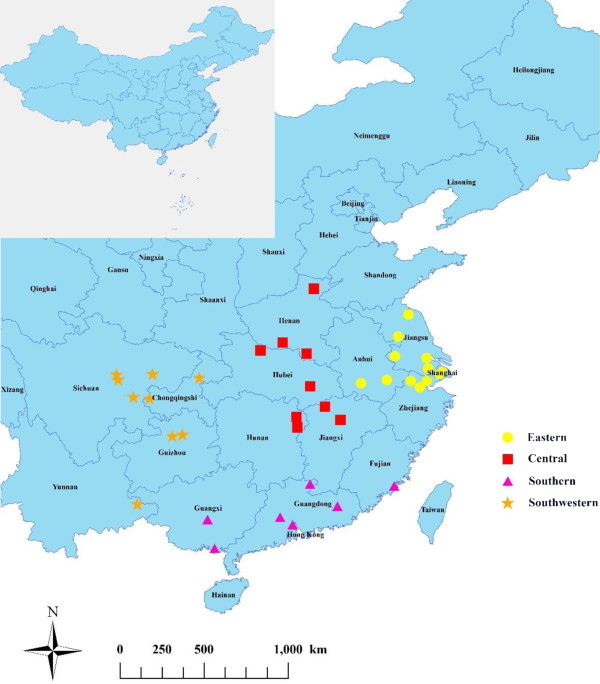
Distribution of 36 selected study communities in subtropical China.

Thirty-six communities in subtropical China affected by the 2008 cold spell were selected for this study. These communities were all selected from the China’s Disease Surveillance Points system (DSPs) according to mortality data quality (mortality > 0.6%) and population size (>200,000) in order to assure enough daily death counts for community-specific time series analysis. Of the 36 communities, 12 communities were in urban areas, and the others in rural areas. They are distributed across 4 geographical regions: eastern China (Jiangsu Province, Zhejiang Province, Anhui Province and Shanghai Municipality), central China (Henan Province, Hubei Province, Hunan Province and Jiangxi Province), southern China (Fujian Province, Guangdong Province and Guangxi Province) and southwestern China (Sichuan Province, Guizhou Province, Yunnan Province and Chongqing Municipality). The 4 regions include 15 provinces/municipalities, home to 784 million inhabitants. The number of study communities in each region was 12, 8, 7 and 9, respectively (Figure 1).

### Data collection

Daily non-accidental mortality data from each study community were obtained from the Chinese Center for Disease Control and Prevention for the period December to March of 2006–2010. The original data was from death certificates, which included the causes and dates of death, gender, age, and place of death (hospital wards, emergency rooms and home). Non-accidental causes of deaths were categorized using codes A00–R99 from the International Classification of Diseases 10th Revision (ICD–10) [19]. The codes J00–J99, I00-I99 and I60-I69 represent respiratory diseases (RESP), cardiovascular diseases (CVD), and cerebrovascular diseases (CBD), respectively. Demographic data in each community were obtained from the sixth national population census which was conducted in 2010 [20].

The corresponding daily meteorological data were collected from the China Meteorological Administration and included latitude, daily average temperature (Tm), maximum temperature (Tmax), minimum temperature (Tmin), average wind speed (WS) and relative humidity (RH).

### Study duration and definition of cold spell

In order to exclude the influence of high temperature on mortality, only winter seasons (December to February) in 2006–2010 were included in this study. The 2008 cold spell started in the middle of January, and ended in the middle of February. In order to estimate the expected long lag effects [3,21], March was also included in the study.

Definitions of cold spell vary greatly across different studies [4,11,22,23]. For instance, Kysely et al. defined a cold spell as “a period of days on which air temperature did not exceed -3.5°C” [4], and Hickey suggested that “a cold spell could consist of a period of 10 consecutive days when the minimum air temperature was 5°C or more below normal” [23]. In our previous study, we defined “a weather fluctuation as a cold spell if the minimum daily temperature fell below the 5th percentile of temperatures recoded at that community from January 2006 through December 2009 for at least 5 consecutive days” [3]. However, some researchers have argued that it may be more appropriate to use daily average temperature to define a cold spell because it reflects the exposure throughout the whole day, while minimum or maximum temperature only represent a short period. Furthermore, daily average temperature can be easily interpreted for decision making purposes [16,24]. Therefore, in the current study, a cold spell was defined as five or more consecutive days with daily average temperature falling below the 5th percentile of daily mean temperatures recorded at each community from December to March of 2006–2010. According to this definition, study days were divided into three groups: 2008 cold spell days, cold spell days in 2006, 2007, 2009 and 2010, and non-cold spell days.

### Statistical analysis

#### ***Estimation of community-specific excess risk of the 2008 cold-spell***

The association between the 2008 cold spell and mortality was estimated using Poisson regression with a distributed lag non-linear model (DLNM) [25,26], which can be written as

(1) $$\begin{array}{ll} \text{Log}\left[E\left(Y_{t}\right)\right] & =\;\alpha \;+\beta T_{t,l}\left(Z\right)+\mathrm{ns}\left(\mathrm{RH},\;\mathrm{df}\right)+\mathrm{ns}\left(\mathrm{WS},\;\mathrm{df}\right)+\mathrm{ns}\left(\text{year},\;\mathrm{df}\right)+\mathrm{ns}\left(\text{month},\;\mathrm{df}\right)\\ & \;+\;\mathrm{ns}\left(\mathrm{day},\;\mathrm{df}\right)\\ & \;+\;\eta \text{DOW}\\ & =\;\alpha \;+\beta T_{t,l}\left(Z\right)+\;\text{COVs} \end{array}$$

Where *t* is the day of observation; *E*(*Y*_*t*_) is the expected number of deaths on day *t*; Z represents cold spell exposure, which was defined as a categorical variable (2 = 2008 cold spell days, 1 = cold spell days in 2006, 2007, 2009 and 2010, and 0 = non-cold spell days). “Non-cold spell days” were defined as the reference group for calculating relative risks (RR). *T*_*t,l*_ is a matrix obtained by applying the DLNM to cold spell; *β* is the vector of coefficients for *T*_*t,l,*_ and *l* is the number of lag days*.* We employed a B-spline function and a natural cubic spline function to estimate the non-linear and lagged effect of cold spell, respectively. To completely capture the overall effects of the 2008 cold spell exposure, a lag structure of up to 27 days was fitted, which is consistent with previous studies [14,21,27]. Degrees of freedom (*df*) for the lag structure were chosen based on Akaike information criterion (AIC) [28]. It was found that 2 *df*s for non-linear and 5 *df*s for the lag produced the best model fitting. *ns*() is a natural spline. Both *df*s for RH and WS were set to 3, consistent with previous studies [13,21,29]. Another 3 *df*s were used to smooth year, calendar month and day to control for secular and seasonal trends. DOW is a dummy variable representing day of the week, and η is vector of coefficients. COVs represents all other covariates in the model. In this model, excess risks (ER) were reported, which indicated the percentage of increased death risk due to exposure to the 2008 cold spell compared to the non-cold spell. The cumulative effect of the 2008 cold spell on mortality during the lag 0–27 days was defined as cumulative excess risk (CER) [25].

#### ***Estimation of community-specific main and added effects of the 2008 cold-spell***

As demonstrated by Gasparrini et al., the effect of heat waves can be divided into the main effect induced by the independent effects of daily temperature levels, and an added effect related to the duration of high temperatures sustained for several consecutive days [12]. Huang et al. also employed this method to estimate the added effect of cold spell on CVD mortality in Brisbane [30]. In this study, we also estimated the main and added effects of the 2008 cold spell on mortality. Another DLNM was employed, which is expressed as:

(2) $$\begin{array}{ll} \text{Log}\left[E\left(Y_{t}\right)\right]\;= & \alpha \;+\beta_{m}T_{\mathrm{mt},l}\left(Z_{m}\right)+\beta_{a}T_{\mathrm{at},l}\left(Z_{a}\right)\\ & +\;\text{COVs} \end{array}$$

Z_m_ and Z_a_ represent the variables for the main and added effects of the 2008 cold spell; *T*_*mt,l*_ and *T*_*at,l*_ are two matrixes obtained by applying the DLNMs to the main and added effects of the 2008 cold spell, while *β*_*m*_ and *β*_*a*_ are vectors of coefficients for *T*_*mt,l*_ and *T*_*at,l*_, respectively. *l* is the number of lag days*.* We set both the maximum lag duration of the main and added effects of the 2008 cold spell as 27 days. We employed a B-spline function to estimate the non-linear main effects, and natural cubic spline functions to estimate the lagged main and added effects of the 2008 cold spell. According to the AIC principles [28], *df* of 5 was employed for the B-spline function of the main effect. Both *df*s of 5 were set for natural cubic spline functions of the main as well as added effects. The main effect was estimated by predicting the relative risk for median temperature among 2008 cold spell days versus the 50th percentile of temperature among non-cold spell days. This reference was chosen as a temperature at which little if any adverse effect of temperature on mortality is expected. Before estimating the added effect of the 2008 cold spell, the variable was coded as a dichotomous variable, 0 indicating non-cold spell days and the first day of the 2008 cold spell, and 1 indicating the remaining days of the 2008 cold spell. Therefore, the added effect was computed by estimating the relative risk of 1 compared to 0 [12]. Other parameter settings were the same as in equation (1).

#### ***Summary effects of the 2008 cold spell on mortality***

A series of Bayesian meta-analysis models were used to estimate the summary effects of the 2008 cold spell on mortality in all study communities and different regions [31,32]. The combined CER of the 2008 cold effect among different communities was defined as summary CER. Multivariate meta-analysis models were performed to estimate the summary lag structure of cold effects.

#### ***Calculation of excess deaths related to the 2008 cold spell***

According to the sixth national population census [20] and method recommended by World Health Organization (WHO) [33], the number of excess deaths in each geographical region was calculated by the following formula:

(3) ED=ER×P×n×N

where ED is the number of excess deaths attributable to the 2008 cold spell; ER is the cumulative ER of the 2008 cold spell on total mortality; P represents average daily mortality during non-cold spell days; n is the number of 2008 cold spell days, and N is the total population in each geographical region. The total number of excess deaths induced by this cold spell was calculated as the sum of excess deaths from 4 geographical regions.

#### ***Sensitivity analysis***

A series of sensitivity analyses were performed to test the robustness of our results. We changed degrees of freedom for year (2–4) and calendar month (2–3). When estimating the summary CER of 2008 cold spell on mortality, the largest CER, the smallest CER and both in all communities were respectively removed from the meta-analyses. In addition, we also changed meta-analysis methods from a random effects model to a fixed effects model.

All statistical tests were two-sided, and p <0.05 was considered statistically significant. We used R software (version 2.15.2; R Development Core Team 2012, http://www.R-project.org/) to analyze the data. The “dlnm” package was used to fit Poisson regression [25]. The “Metafor” package was used to fit Meta-analysis [31]. According to the aims of the present study, we only report the excess mortality related to the 2008 cold spell. The effect of cold spell in 2006, 2007, 2009 and 2010 was shown in Additional file 1.

## Results

**Figure 2:**
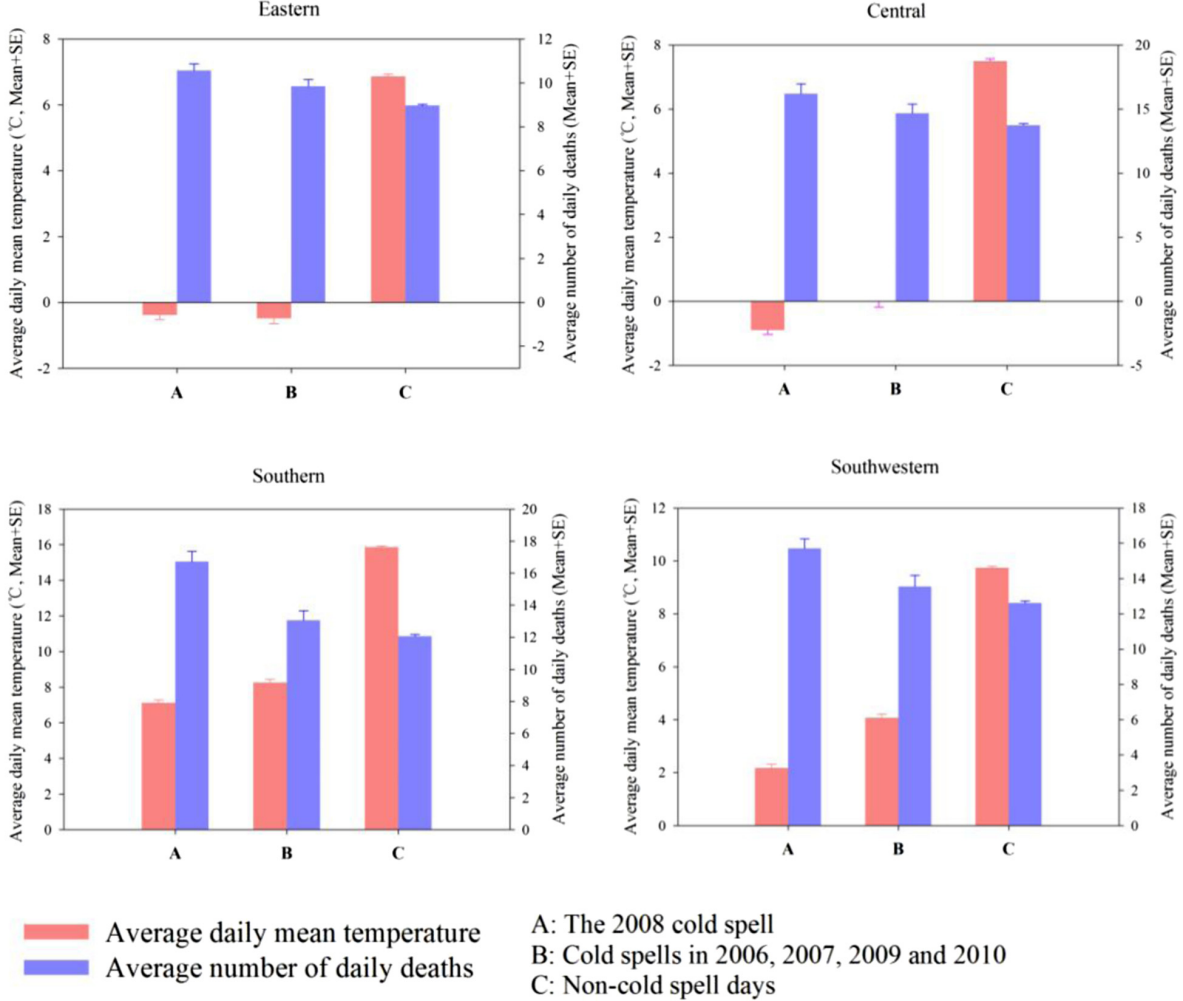
Average daily mean temperature and number of daily deaths in the 2008 cold spell, cold spells in other studied years (2006, 2007, 2009 and 2010) and non-cold spell days, in four geographical regions of subtropical China.

Table 1 presents the distributions of mortality, latitude and weather statistics during the 2008 cold spell, the cold spells in 2006, 2007, 2009 and 2010, and non-cold spell days in 36 communities. All the communities are located between 27.3 and 36.0 degrees north. The 2008 cold spell persisted from 14 to 36 days in the 36 communities, with an average duration of 26.5 days. Compared to cold spells in other years as well as non-cold spell days, the 2008 cold spell had a higher mortality and relative humidity (RH), and lower temperatures. Similar results were also observed in each geographical region (Figure 2).

**Table 1:** Mean and specific percentiles for studied variables in December to March 2006–2010 in 36 study communities of subtropical China

	**Mean**	**Min**	**25th**	**75th**	**Max**
*All days*					
Total mortality	11.7	2.9	6.6	15.8	26.8
CVD	5.1	1.1	2.8	7.1	13.7
RESP	2.3	0.4	1.3	3.5	6.7
CBD	2.6	0.5	1.4	3.0	7.1
Latitude (degree north)	29.1	21.3	27.5	31.5	36.0
Mean temperature (°C)	8.9	0.9	6.4	10.1	16.9
Minimum temperature (°C)	5.7	-2.3	3.1	7.4	14.2
Maximum temperature (°C)	13.3	4.4	11.0	14.2	21.0
Wind speed (m/s)	2.0	0.8	1.3	2.6	5.8
Relative humidity (%)	72.8	60.2	70.2	75.4	82.3
*2008 cold spell days*					
Total mortality	14.6	2.3	9.0	19.1	36.1
CVD	6.6	0.8	3.6	9.5	19.5
RESP	3.1	0.2	2.0	3.9	9.1
CBD	3.3	0.3	2.0	3.8	10.3
Latitude (degree north)	29.1	21.3	27.5	31.5	36.0
Mean temperature (°C)	1.5	-8.0	-0.8	3.8	8.8
Minimum temperature (°C)	-0.4	-11.3	-2.6	2.2	6.8
Maximum temperature (°C)	3.9	-4.3	1.8	6.4	12.1
Wind speed (m/s)	2.0	0.7	1.4	2.5	4.7
Relative humidity (%)	76.7	65.0	73.4	80.3	91.4
Days of cold spell	26.5	14.0	22.0	32.0	36.0
*Cold spell days in 2006, 2007, 2009, 2010*					
Total mortality	12.6	3.8	6.9	18.3	27.3
CVD	5.6	1.2	3.4	7.1	14.7
RESP	2.6	0.6	1.4	3.2	6.4
CBD	2.7	0.6	7.4	1.2	3.3
Latitude (degree north)	29.1	21.3	27.5	31.5	36.0
Mean temperature (°C)	2.3	-8.0	-0.1	5.0	9.3
Minimum temperature (°C)	-0.2	-10.4	-3.2	3.3	7.9
Maximum temperature (°C)	5.9	-4.7	3.2	7.7	13.2
Wind speed (m/s)	2.3	0.9	1.5	2.7	5.2
Relative humidity (%)	71.2	53.1	62.0	79.2	83.9
Days of cold spell	19.4	6.0	15.0	25.0	34.0
*Non cold spell days*					
Total mortality	11.6	2.8	6.5	15.5	26.6
CVD	5.0	1.1	13.4	2.8	6.9
RESP	2.3	0.4	1.2	3.5	6.7
CBD	2.5	0.5	1.4	3.0	7.0
Latitude (degree north)	29.1	21.3	27.5	31.5	36.0
Mean temperature (°C)	9.4	1.4	7.1	10.5	17.6
Minimum temperature (°C)	6.1	-1.9	3.6	7.8	14.8
Maximum temperature (°C)	14.0	4.9	11.8	14.7	21.9
Wind speed (m/s)	2.0	0.8	1.3	2.6	5.8
Relative humidity (%)	72.7	60.1	70.2	75.4	81.8

**Figure 3:**
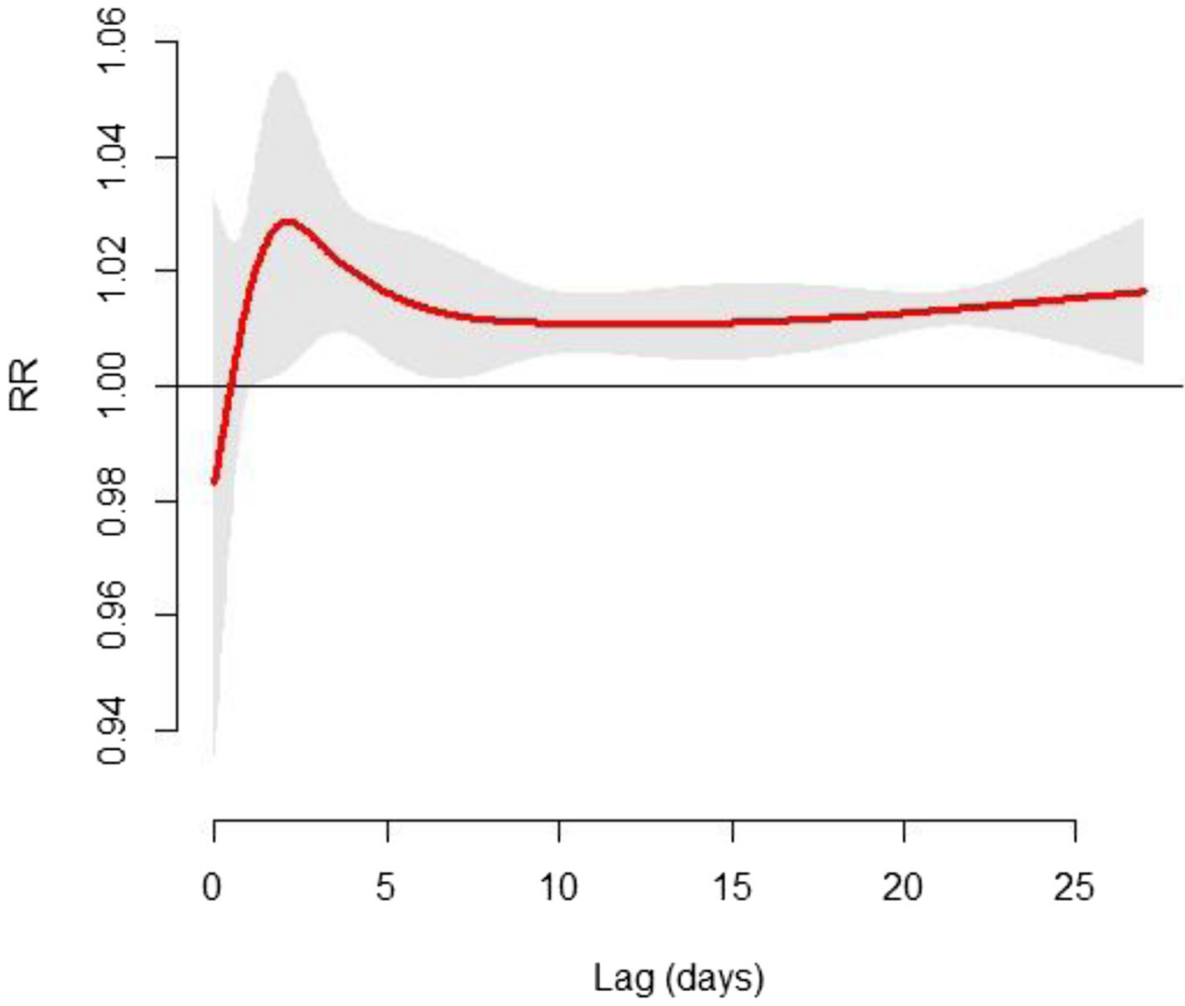
**Multivariate meta-analysis based summary single day RRs (95% CI) of the 2008 cold spell on non-accidental mortality during lag 0–27 days in 36 study communities of subtropical China.***Note*: RRs are adjusted for secular trend, wind speed, day of week and relative humidity.

**Figure 4:**
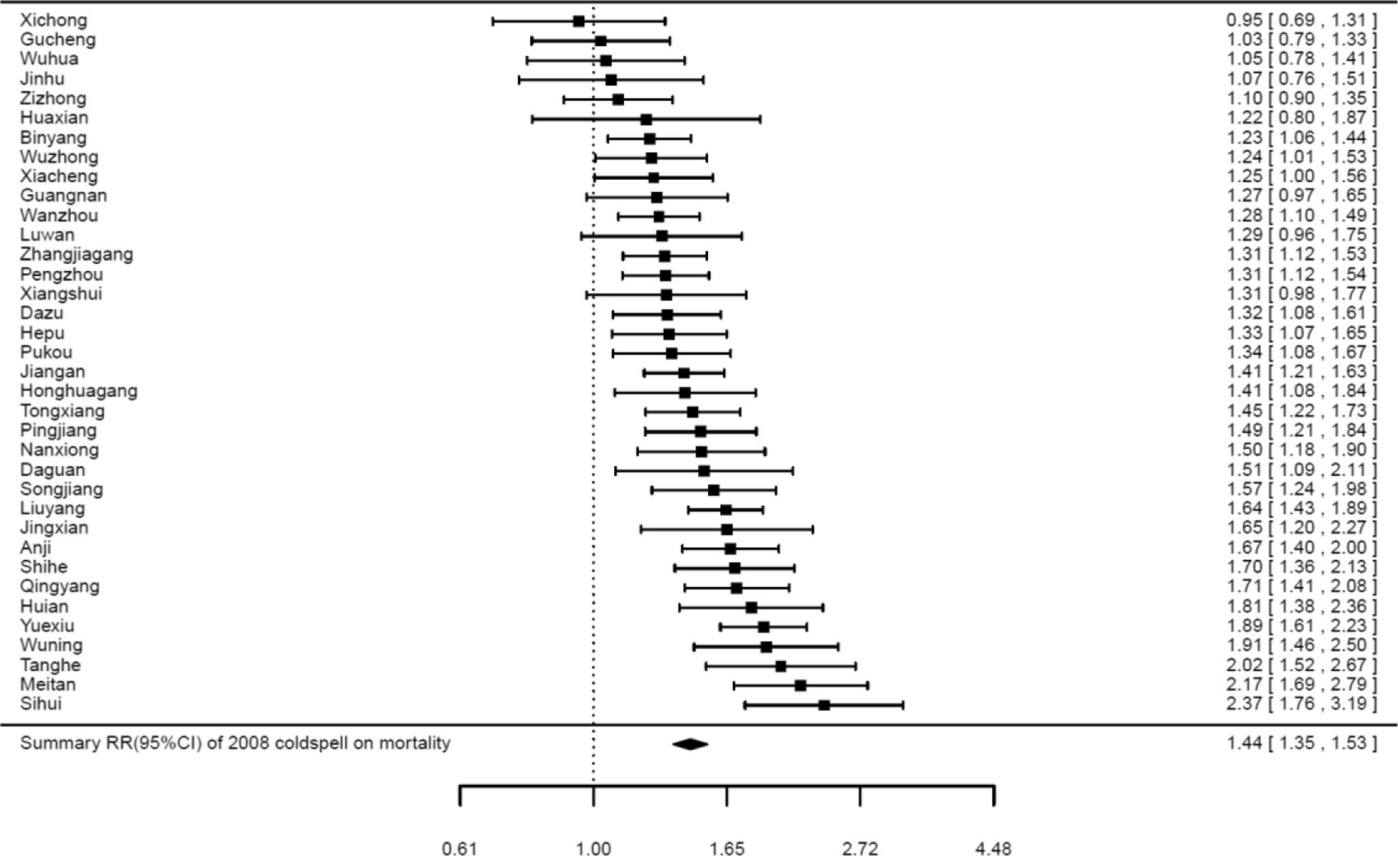
**Bayesian-based summary RR (95% CI) of the 2008 cold spell on total non-accidental mortality for lag 0–27 days in 36 study communities across subtropical China.***Note*: All results in every community were adjusted for secular trend, wind speed, day of week and relative humidity.

**Figure 5:**
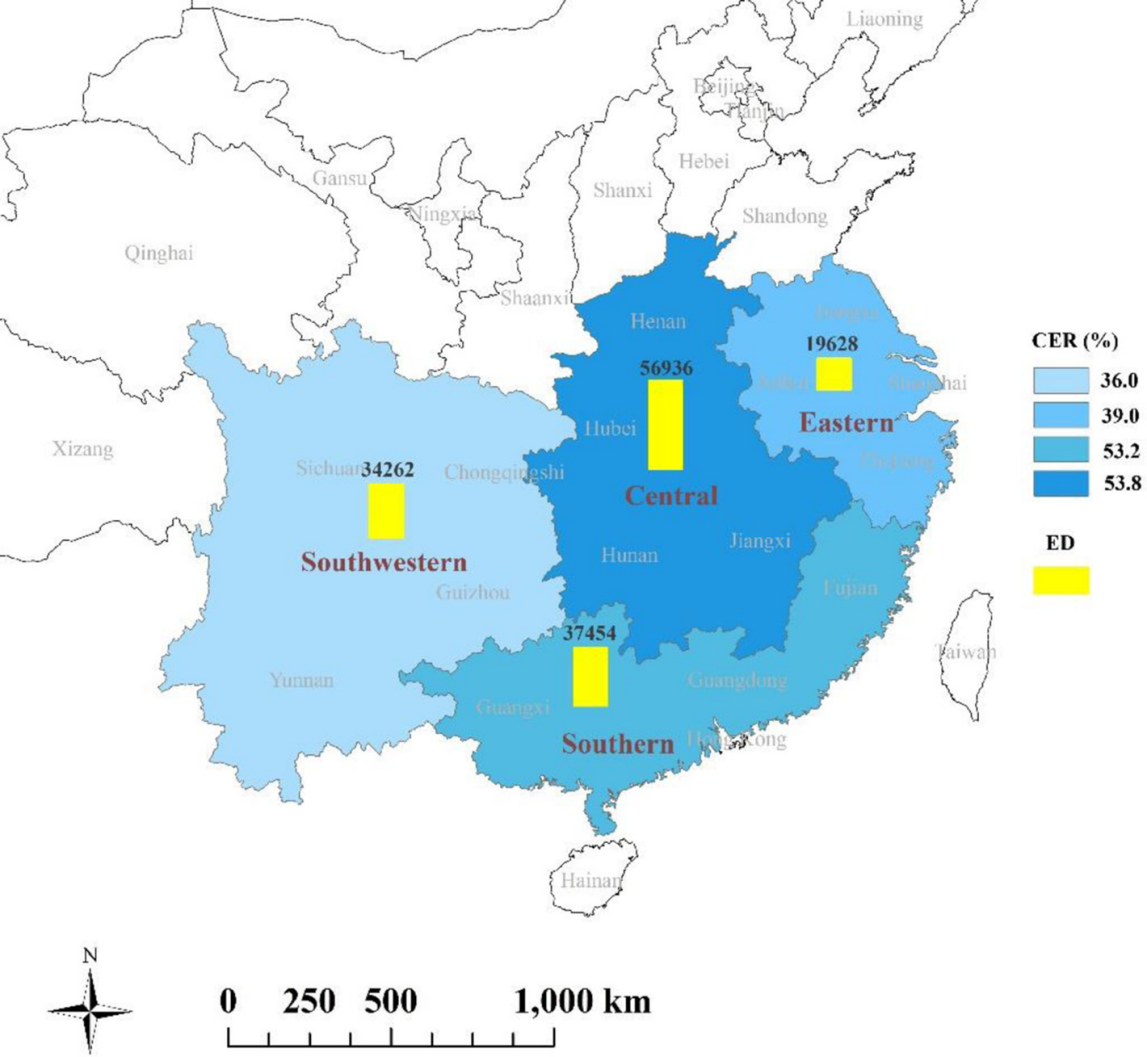
**Cumulative excess risks (CER, %) and excess deaths (ED) of the 2008 cold spell on non-accidental mortality during lag 0–27 days in four geographical regions of subtropical China.***Note*: All results were adjusted for secular trend, wind speed, day of week and relative humidity.

Figure 3 illustrates the lag structure for the effect of the 2008 cold spell on non-accidental mortality for all 36 communities. The mortality risk increased to a maximum after three days exposure to the cold spell, approached 1.01 after 7 days, and then leveled off for the next 3 weeks. Similar patterns were also observed in southern, eastern and central China. However, the lag structure during the first week was different in Southwestern China. It rapidly declined in the first 2 days, and then increased to around 1.02 of RR at lag 7 days (Additional file 2). Figures 4 and 5 show the cumulative excess mortality (CER) of the 2008 cold spell on mortality at lag 0–27 days. There was a total of 43.8% (95% CI: 34.8% ~ 53.4%) excess deaths caused by the 2008 cold spell compared to non-cold spell days in all 36 communities, and higher death risks were observed in southern China (CER = 53.2%, 95% CI: 33.1% ~ 76.4%) and central China (CER = 53.8%, 95% CI: 26.1% ~ 87.6%) compared to southwestern China (CER = 36.0%, 95% CI: 17.6% ~ 57.4%) and eastern China (CER = 39.0%, 95% CI: 29.5% ~ 49.2%). The attributable numbers of deaths were 19,628, 56,936, 37,454, and 34,262 in eastern, central, southern and south western China, respectively. In total, 148,279 excess deaths were associated with the 2008 cold spell across 15 subtropical provinces of China.

Estimated CERs after stratification by demographic characteristics, location, causes of death and place of death are provided in Table 2. They were higher for RESP (CER = 61.9%, 95% CI: 41.5% ~ 85.3%) than for CVD (CER = 52.9%, 95% CI: 42.1% ~ 64.5%) or CBD (CER = 54.3%, 95% CI: 36.5% ~ 74.4%), and for females (CER = 48.9%, 95% CI: 38.6% ~ 60.1%) than for males (CER = 38.5%, 95% CI: 29.4% ~ 48.3%). The CERs were statistically significant for all age groups, and much higher for the elderly (CER = 65.4% for population ≥ 85 years compared to CER = 17.9% for those aged 0–64 years). The risk of people dying in emergency rooms was significantly higher (CER = 80.3%, 95% CI: 27.9% ~ 154.3%) than the risk of dying in hospital wards (CER = 22.9%, 95% CI: 5.7% ~ 42.9%) or at home (CER = 44.8%, 95% CI: 35.5% ~ 54.7%). CERs in rural areas were higher than in urban areas for RESP (66.0% versus 53.7%) and CBD (61.1% versus 39.0%), but the reverse for CVD (46.8% versus 65.7%).

**Table 2:** Summary of cumulative excess risks (CER, %) of the 2008 cold spell on non-accidental mortality at lag 0–27 in 36 communities of subtropical China, by cause of death, age, gender and place of death

**Non-accidental mortality**	**All samples**	**Location**	
		**Urban**	**Rural**
	**Summary CER (95% CI)**	**Summary CER (95% CI)**	**Summary CER (95% CI)**
All	43.8*(34.8 ~ 53.4)	46.0*(33.7 ~ 59.5)	42.7*(30.3 ~ 56.4)
Cause of death			
CVD	52.9*(42.1 ~ 64.5)	65.7*(48.0 ~ 85.4)	46.8*(34.0 ~ 60.9)
RESP	61.9*(41.5 ~ 85.3)	53.7*(20.4 ~ 96.2)	66.0*(40.7 ~ 95.7)
CBD	54.3*(36.5 ~ 74.4)	39.0*(22.0 ~ 58.3)	61.1*(35.7 ~ 91.2)
Age groups (years)			
0-64	17.9*(7.7 ~ 29.1)	19.6*(5.7 ~ 35.2)	18.2*(4.6 ~ 33.6)
65-74	41.8*(29.6 ~ 55.3)	34.0*(18.9 ~ 50.9)	46.1*(29.5 ~ 65.0)
75-84	46.9*(34.6 ~ 60.3)	55.6*(32.8 ~ 82.2)	42.3*(28.3 ~ 57.9)
≥85	65.4*(51.3 ~ 80.9)	68.1*(46.1 ~ 93.5)	63.1*(44.7 ~ 83.9)
Gender			
Male	38.5*(29.4 ~ 48.3)	39.5*(24.9 ~ 55.8)	38.1*(26.3 ~ 51.0)
Female	48.9*(38.6 ~ 60.1)	52.8*(40.1 ~ 66.6)	46.8*(32.1 ~ 63.1)
Place of death			
Wards of hospital	22.9*(5.7 ~ 42.9)	20.9 (-1.8 ~ 48.8)	26.5*(1.6 ~ 57.5)
Emergency room	80.3*(27.9 ~ 154.3)	133.6*(49.1 ~ 265.8)	38.3 (-16.7 ~ 129.5)
Home	44.8*(35.5 ~ 54.7)	39.0*(26.2 ~ 53.2)	46.5*(34.8 ~ 59.3)

*Note*: All results in every community were adjusted for secular trend, wind speed, day of week and relative humidity.

*P < 0.05.

Table 3 shows the main effect and added effect of the 2008 cold spell on non-accidental mortality at lag 0–27 days. The main effect (CER = 22.5%, 95% CI: 5.2% ~ 42.6%) was much larger than the added effect (CER = 9.4%, 95% CI: -7.8% ~ 29.9%). This phenomenon was also observed in eastern China (CER = 30.5% versus 4.6%), central China (CER = 45.1% versus 11.2%) and southern China (CER = 20.6% versus 13.7%), but not in southwestern China (CER = 8.1% versus 15.2%).

**Table 3:** Summary of the main effect and added effects of the 2008 cold spell on non-accidental mortality at lag 0–27 in subtropical China

**Non-accidental mortality**	**Main effects**	**Added effects**
	**Summary CER (95% CI)**	**Summary CER (95% CI)**
Eastern	30.5*(6.0 ~ 60.7)	4.6 (-15.1 ~ 28.9)
Central	45.1 (-25.2 ~ 181.5)	11.2 (-44.6 ~ 123.3)
Southern	20.6 (-11.8 ~ 64.8)	13.7 (-23.1 ~ 68.2)
Southwestern	8.1(-38.5 ~ 90.1)	15.2 (-47.1 ~ 150.9)
Total	22.5*(5.2 ~ 42.6)	9.4 (-7.8 ~ 29.9)

*Note*: All results were adjusted for secular trend, wind speed, day of week and relative humidity.

*P < 0.05.

### Sensitivity analyses

Summary CERs of the 2008 cold spell on mortality varied from 37.8% to 43.8% for changes in the *df* for smoothness of year and calendar month (Additional file 3), from 42.2% to 45.1% for the changes of fitness methods in the meta-analyses (Additional file 4), and from 42.2% to 44.6% for different lag days (Additional file 5). These findings indicated that our results were robust.

## Discussion

To understand how extreme low temperatures impact human health is crucial not only for policymakers who develop prevention and intervention strategies for extreme weather events, but also for the community, especially for those where extreme cold spells are uncommon. The 2008 cold spell that occurred in subtropical China provided a unique research opportunity to assess the health impacts of extreme cold spells in subtropical regions. In the present study, we assessed the effects of the 2008 cold spell on mortality, spatial heterogeneity of the effects and demographic modifying factors using data from 36 communities of subtropical China. To our knowledge, this is the first multi-community study to investigate effects of the 2008 cold spell on mortality in subtropical China.

As expected, we found the 2008 cold spell significantly increased mortality risk compared to non-cold spell periods, which was consistent with previous studies [2,4,8,11,34-37]. For example, an average excess mortality of 12.8% was found during the cold spells in 1979–1997 of Netherlands [36]. The cumulative excess mortality of 75+ age people in Moscow were 9.9% and 8.9% during the two cold spells of 2006 [37]. However, we found that the excess mortalities in previous studies were considerably lower than our findings [4,11,36,37], which was also larger than the effect of cold spell in 2006, 2007, 2009 and 2010 (Additional file 1). There are several possible reasons for this difference. Firstly, the intensity and duration of cold spells varied across different studies, which largely modified cold-related effects [11,38]. The 2008 cold spell appears to be more intensive and longer than cold spells in previous studies. For instance, the cold spells in 23 Italian cities in 2012 lasted 6–18 days, and the temperature dropped 3.5-7.9°C compared to the reference days [11]. In our study, the average daily mean temperature during the 2008 cold spell dropped 7.3-8.7°C compared to the non-cold spell days, and the mean duration of the 2008 cold spell was 26.5 days, with a range of 14–36 days across all study communities. Secondly, methods and cold spell definition employed to estimate the excess mortality varied in different studies, which can lead to the difference in findings between studies [4,11,22,23]. For instance, Kysely et al. defined a cold spell as a period of days when air temperature did not exceed -3.5°C, and calculated the expected (baseline) number of deaths using the mean annual cycle smoothed by 15-day running means [4]. Thirdly, populations in subtropical regions may be more sensitive to cold spells and lack necessary preparedness because cold spells are not common in these regions.

Our study confirmed previous findings that health impacts of low temperatures appeared to be larger in warmer regions than in colder regions [2,10,34,35]. Populations in southern and central China were found to be at higher risk than those in the eastern and southwestern China. This regional variation could be explained by intensity and duration of the cold spell, socio-demographic characteristics and adaptive capacity [9,11,35,39,40]. In eastern China, the average temperature in winter was lower than that in the other three regions and people may acclimatize to low temperatures and take more effective adaptive measures against cold temperatures during a cold spell [41]. In contrast, populations in southern China are less well acclimatized to extreme low temperatures and few buildings in this region have heating systems to respond to low temperatures. The reasons for the lower effect of the 2008 cold spell in southwestern China is not clear, and require further study in the future.

We further found that morality risks of the 2008 cold spell were higher for people who died of respiratory illness compared with those where cardiovascular or cerebrovascular illness were the reported causes of death [3], which is consistent with some previous studies [2,10,11,40,41]. This may be due to the more rapid spread of infectious diseases, reduced response mechanisms of the upper respiratory tract, suppressed immune responses, exacerbated chronic respiratory diseases, and increment of fibrinogen concentration related to the respiratory infections [11,38,42]. Further stratification analyses revealed that higher respiratory and cerebrovascular mortality risks were found in rural areas, and higher cardiovascular mortality risks were observed in urban communities. The possible reason for this difference is that people in rural areas are used to staying indoors during winter, and hence are likely to have higher exposure to a complex pollutant mixture of particulate matter (PM) and other toxic compounds [43] associated with cigarette and biomass fuels or coals use for heating and cooking [44]. These pollutants have been proved to be associated with some respiratory and cerebrovascular diseases, such as infections, asthma, chronic obstructive pulmonary disease (COPD) [43], hypertension [45], and strokes [46]. In addition, the less air condition use in rural area could increase people’s vulnerability to extremely cold weather [47]. The reasons for higher CVD mortality risks in urban areas may be partially attributable to the higher prevalence of coronary heart diseases in urban areas of China [48]. Extreme low temperature exposure could increase the cardiovascular stress through changes in blood pressure, vasoconstriction, increase in blood viscosity and levels of red blood cell count, plasma cholesterol and plasma fibrinogen [4,38,42], hence increasing the risk of death for people with chronic cardiovascular diseases during the cold spell.

In the current study, we confirmed that age was another important modifier of the temperature-mortality relationship [2,10]. The elderly were more susceptible to low temperatures than younger people, and this can be attributed to factors such as limited thermoregulation and concomitant chronic disease [11]. In addition, we found that place of death with the highest risk was emergency rooms in urban cities, while in rural areas, the place of death with the highest risk was home. The possible reason is that in urban areas, people are sent to an emergency room quickly and with more severe diseases during an extreme cold spell, while people in rural areas are unable to access emergency services when they are ill. In addition, influenced by the Chinese tradition, people who are almost dying in rural areas tend to stay at home rather than go to hospital. These results indicate that increasing access to emergency services and early preparedness for health facilities during extreme cold days are crucial for preventing cold-related health effects in rural areas.

We also identified that females had higher mortality risk than males, a finding similar to some previous studies [35,39,49]. This phenomenon may be mainly due to a higher proportion of women in the elderly because women have a higher life expectancy than men in China, leading to a much larger proportion of vulnerable women [20]. In addition, males usually have better thermoregulation ability than females due to the physiological differences [50], such as sex hormones, body water regulation and exercise capacity [51]. However, some other studies found inverse effects between males and females [40,41], indicating that there may be some other specific reasons behind sex, such as cultural practices, daily activities and exposure level and hence more studies of this differential vulnerability may be required in the future.

Few previous studies assessed the number of excess deaths of an extreme weather event [12,25,52]. This study observed that 148,279 deaths were attributable to the 2008 cold spell in subtropical China. However, the health effect of the 2008 cold spell was underestimated because we did not include morbidity (including injury) due to this event [13,14]. Our findings found that health impacts of the 2008 cold spell were very large, suggesting that adapting to health effects of cold spells in subtropical regions is necessary in the context of future climate change.

In order to deepen our understanding of the effects of the 2008 cold spell, we further estimated the main and added effects of the 2008 cold spell. We found that cold effects were mostly attributable to extreme low temperatures rather than any added effect related to sustained duration of the low temperatures, which is similar to Huang et al.’s finding that added effects may contribute small proportions in the total effects of cold spells on CVD mortality in Brisbane [30]. This finding suggests that it is the initial cold ‘snap’ that is the main effect and that as the event continued over time people took protective action. This implies that it is necessary to evaluate potential additional effects from cold spells by decomposing the cold effect into a temperature term and an added effect term so we can better understand the mechanism of the cold effects and better plan public health interventions or better estimate the future burden of temperature-related deaths under predicted climate change scenarios.

Some limitations of this study should be mentioned. Firstly, the meteorological variables used were a simple mean of data collected from various monitoring stations, and the variance of measurement may differ from station to station, which may induce larger heterogeneity of the results. Secondly, air pollutants were not adjusted for in this study, which may cause overestimation of the cold effects. Finally, mortality fluctuations in the winter season can partially be attributed to seasonal patterns of illness such as influenza [53]. Unfortunately, influenza data were not available in this study. However, our previous study conducted in Guangdong Province of China indicated that the association between the 2008 cold spell and mortality was not significantly changed after adjusting for influenza death [3].

## Conclusions

The 2008 cold spell significantly increased mortality in subtropical China, which was mainly explained by independent effects of low temperature rather than consecutive days of cold periods. The cold effect was spatially heterogeneous and modified by individual-specific factors. Considering increased weather variability and changes in frequency and intensity of extreme weather events in light of climate change in the future, the findings of the current study imply that it is necessary to develop adaptive plans for cold spells even in subtropical regions, where populations are generally most acclimatized to hot weather both biologically and behaviorally but more sensitive to cold weather due to lower adaptive capacity.

## Abbreviations

CHINAs: Climate and health impact national assessment study; DLNM: Distributed lag linear non-linear model; RESP: Respiratory mortality; CVD: Cardiovascular; CBD: Cerebrovascular mortality; IPCC: The intergovernmental panel on climate change; DSPs: China’s disease surveillance points system; ICD–10: International classification of diseases 10th revision; Tm: Daily average temperature; Tmax: Maximum temperature; Tmin: Minimum temperature; WS: Average wind speed; RH: Relative humidity; RR: Relative risks; CI: Confidence internal; AIC: Akaike information criterion; DOW: Day of the week; COV: Covariate variables; ER: Excess risks; CER: Cumulative excess risk; WHO: World health organization; ED: Excess death; PM: Particulate matter; COPD: Chronic obstructive pulmonary disease.

## Competing interests

The authors declare they have no actual or potential competing financial interests.

## Authors’ contributions

MGZ participated in the design of this study and drafted the manuscript. LJW participated in the design of this study, collected the data and analyzed the data. TL participated in the design of this study, analyzed the data and drafted the manuscript. YHZ participated in the design of this study. HLL participated in the design of the study and draft revision. YL collected the data and analyzed the data. JPX collected the data and analyzed the data. WLZ collected the data and analyzed the data. XFW provided some data. YWZ collected the data. XG collected the data. SR participated in the design of the study and draft revision. CC participated in the design of the study and draft revision. WJM conceived of the study, and participated in its design and coordination. All authors read and approved the final manuscript.

## Supplementary Material

Additional file 1 Summary of cumulative excess risks (CER, %) of the cold spells in 2006, 2007, 2009 and 2010 on non-accidental mortality at lag 0–27 in 36 communities of subtropical China, by cause of death, age, gender and place of death. Click here for file

Additional file 2 **Summary single day RRs (95% CI) of the 2008 cold spell on non-accidental mortality along lag 0–27 days in four geographical regions of subtropical China.***Note*: All results were adjusted for secular trend, wind speed, day of week and relative humidity. Click here for file

Additional file 3 **Sensitivity analyses of *df *** **per year/month on the associations between 2008 cold spell and mortality along lag 0–27 days in 36 communities of subtropical China.***Note*: All results were adjusted for secular trend, wind speed, day of week and relative humidity. Click here for file

Additional file 4 **Sensitivity analyses of meta analysis methods on the association between 2008 cold spell and all mortality along lag 0–27 days in 36 communities of subtropical China.***Note*: All results were adjusted for secular trend, wind speed, day of week and relative humidity. A: Summary CER was estimated by random effect model. B: Summary CER was estimated by fixed effect model. C: Summary CER was estimated by random effect model with removing the largest ER in the total 36 selected communities. D: Summary CER was estimated by random effect model with removing the smallest ER in the total 36 selected communities. E: Summary CER was estimated by random effect model with simultaneously removing the largest and smallest ERs in the total 36 selected communities. Click here for file

Additional file 5 **Sensitivity analyses on the association between 2008 cold spell and all mortality along different lag days in 36 communities of subtropical China.***Note*: All results were adjusted for secular trend, wind speed, day of week and relative humidity. Click here for file
