# Supplementary material for: Health impact of the 2008 cold spell on mortality in subtropical China: the climate and health impact national assessment study (CHINAs)
Source: Environ Health. 2014 Jul 24;13:60. doi: 10.1186/1476-069X-13-60 (PMC4115219; doi:10.1186/1476-069X-13-60)
Supplement: Additional file 1 — Summary of cumulative excess risks (CER, %) of the cold spells in 2006, 2007, 2009 and 2010 on non-accidental mortality at lag 0–27 in 36 communities of subtropical China, by cause of death, age, gender and place of death. [file 1476-069X-13-60-S1.docx]

Additional file 1. Summary of cumulative excess risks (CER, %) of the cold spells in 2006, 2007, 2009 and 2010 on non-accidental mortality at lag 0-27 in 36 communities of subtropical China, by cause of death, age, gender and place of death

| Non-accidental mortality | All samples |
| --- | --- |
|  |  |
|  | Summary CER (95%CI) |
| All | 16.9* (3.2~32.4) |
| Cause of death |  |
| CVD | 22.8* (5.3~43.2) |
| RESP | 36.2* (11.4~66.5) |
| CBD | 21.1 (-0.2~47.1) |
| Age groups (years) |  |
| 0-64 | -11.3 (-24.6~4.3) |
| 65-74 | 20.3* (1.0~43.3) |
| 75-84 | 27.7* (9.2~49.4) |
| ≥85 | 18.2 (-0.4~40.3) |
| Gender |  |
| Male | 13.4 (-1.1~30.0) |
| Female | 17.5* (0.7~37.2) |
| Place of death |  |
| Wards of hospital | 5.1 (-12.4~26.1) |
| Emergency room | 39.1 (-13.8~124.3) |
| Home | 19.2* (1.9~39.4) |

*Note*: All results in every community were adjusted for secular trend, wind speed, day of week and relative humidity.

*: P<0.05
