# Supplementary figures and images for: Health impact of the 2008 cold spell on mortality in subtropical China: the climate and health impact national assessment study (CHINAs)
Source: Environ Health. 2014 Jul 24;13:60. doi: 10.1186/1476-069X-13-60 (PMC4115219; doi:10.1186/1476-069X-13-60)

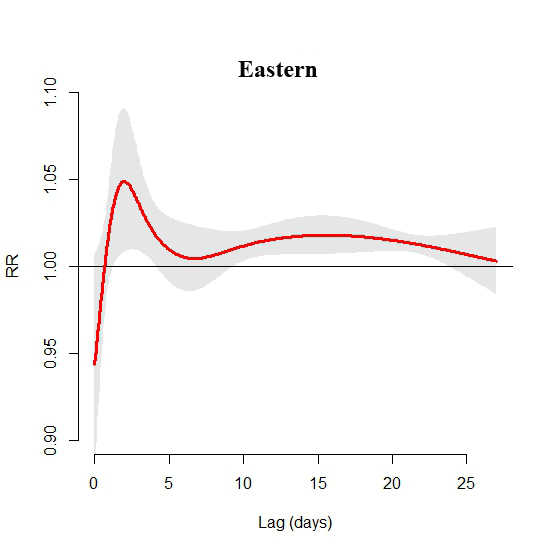

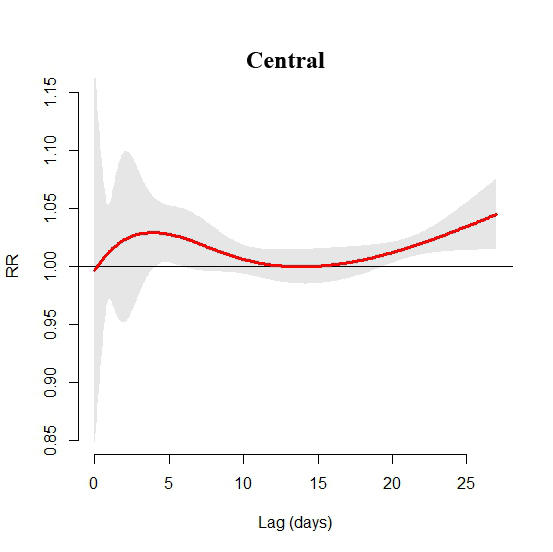


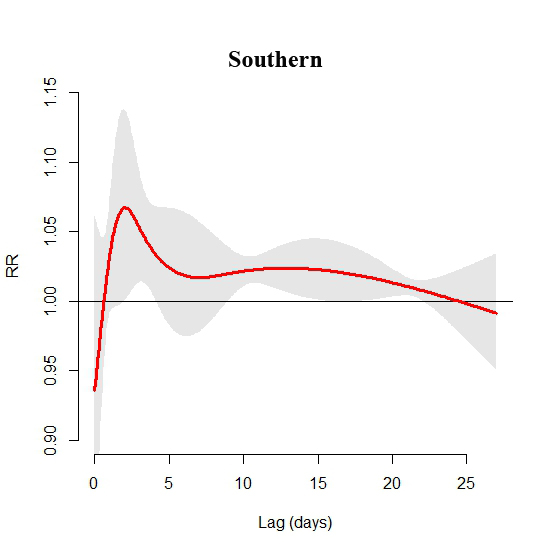

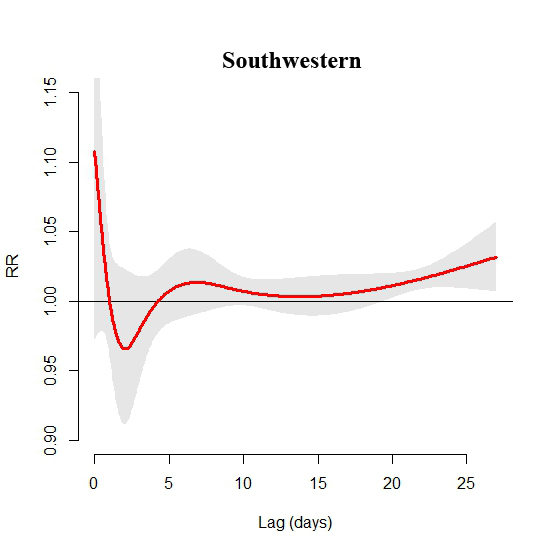

Supplement: Additional file 2 — Summary single day RRs (95% CI) of the 2008 cold spell on non-accidental mortality along lag 0–27 days in four geographical regions of subtropical China.Note: All results were adjusted for secular trend, wind speed, day of week and relative humidity. [file 1476-069X-13-60-S2.docx]

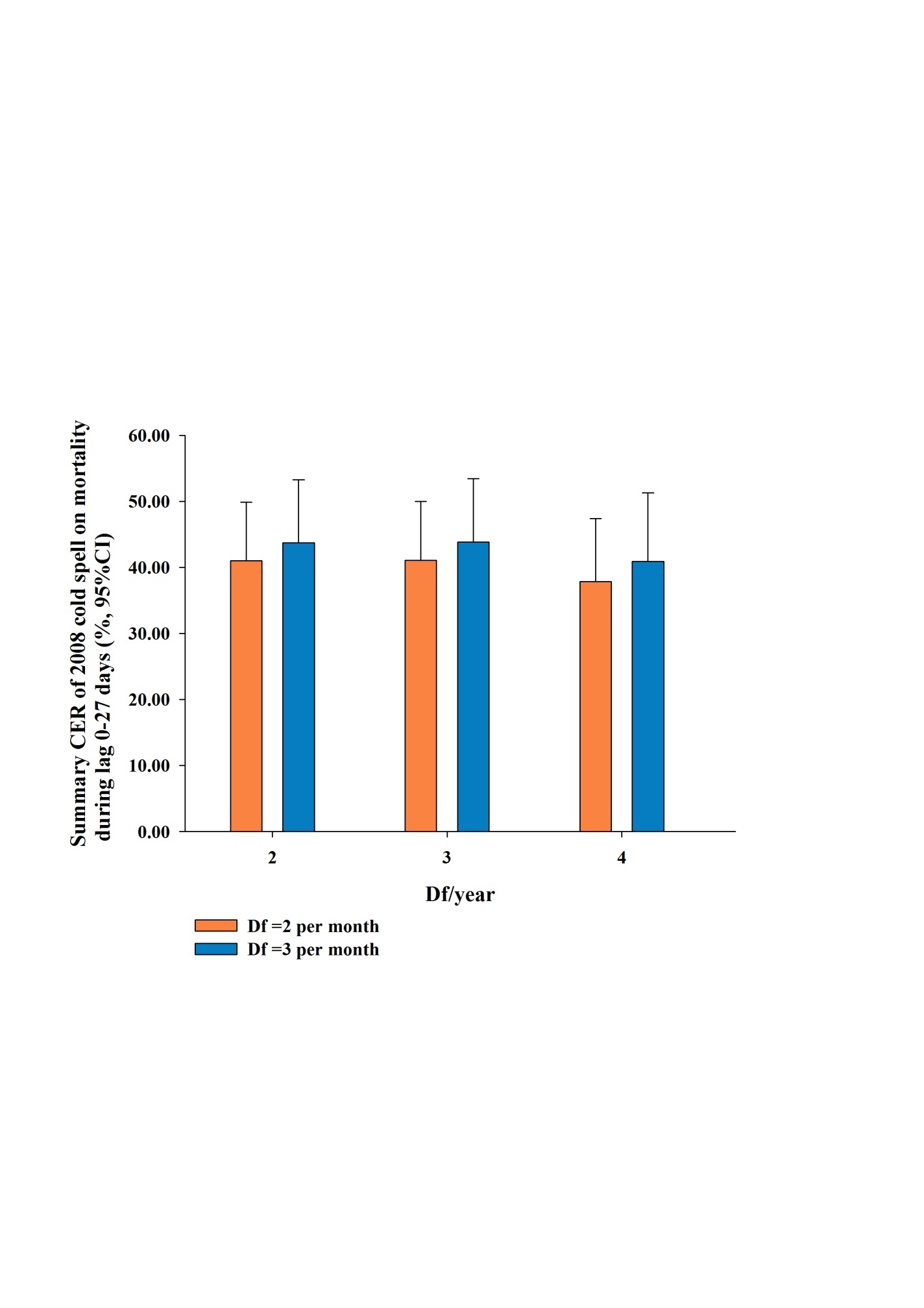

Supplement: Additional file 3 — Sensitivity analyses of df per year/month on the associations between 2008 cold spell and mortality along lag 0–27 days in 36 communities of subtropical China.Note: All results were adjusted for secular trend, wind speed, day of week and relative humidity. [file 1476-069X-13-60-S3.docx]

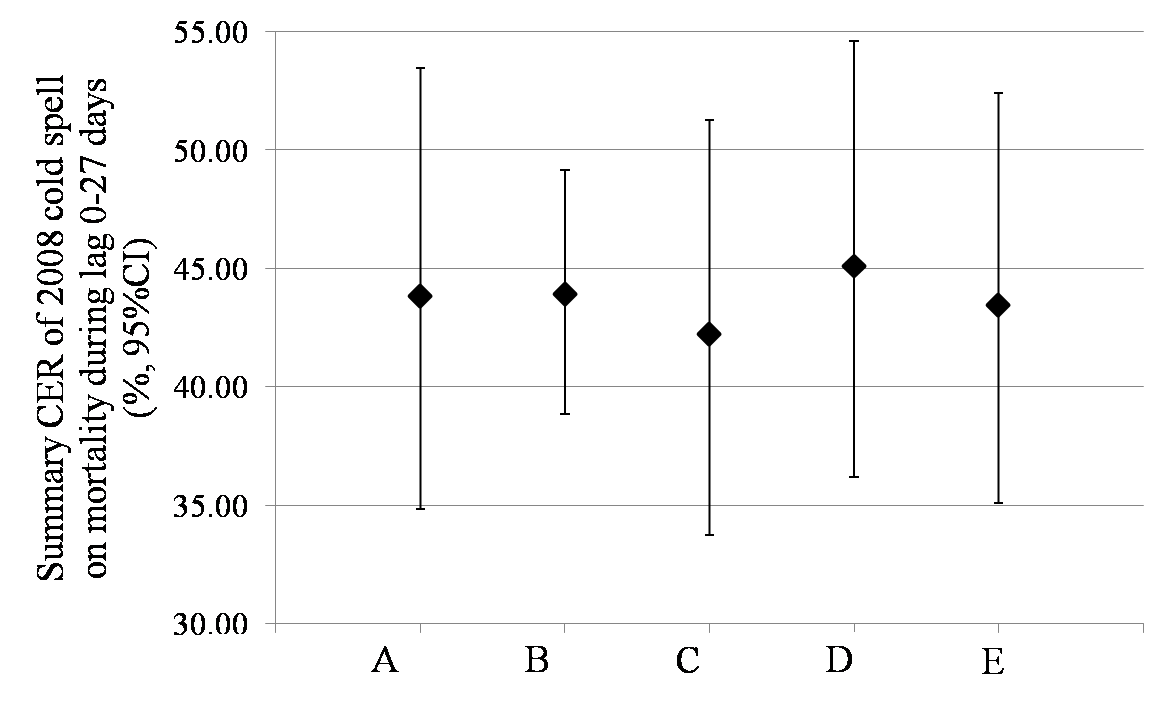

Supplement: Additional file 4 — Sensitivity analyses of meta analysis methods on the association between 2008 cold spell and all mortality along lag 0–27 days in 36 communities of subtropical China.Note: All results were adjusted for secular trend, wind speed, day of week and relative humidity. A: Summary CER was estimated by random effect model. B: Summary CER was estimated by fixed effect model. C: Summary CER was estimated by random effect model with removing the largest ER in the total 36 selected communities. D: Summary CER was estimated by random effect model with removing the smallest ER in the total 36 selected communities. E: Summary CER was estimated by random effect model with simultaneously removing the largest and smallest ERs in the total 36 selected communities. [file 1476-069X-13-60-S4.docx]

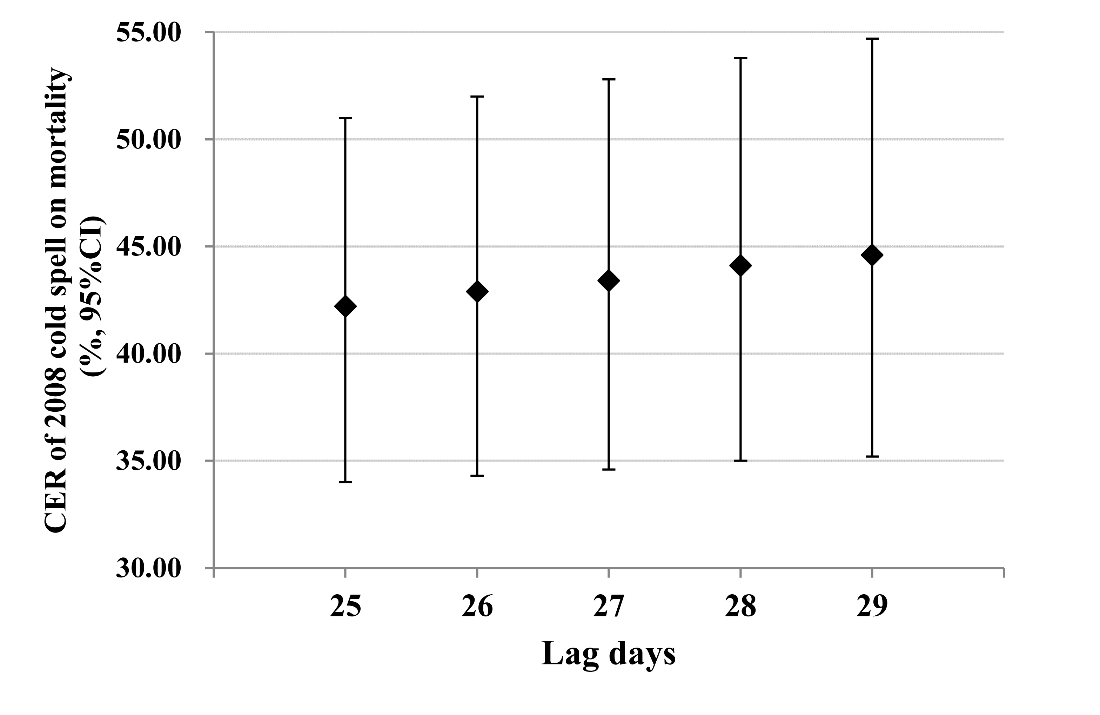

Supplement: Additional file 5 — Sensitivity analyses on the association between 2008 cold spell and all mortality along different lag days in 36 communities of subtropical China.Note: All results were adjusted for secular trend, wind speed, day of week and relative humidity. [file 1476-069X-13-60-S5.docx]
